# Weekly cisplatin for the treatment of patients with ovarian cancer: A protocol for a systematic review of randomized controlled trial

**DOI:** 10.1097/MD.0000000000015001

**Published:** 2019-04-05

**Authors:** Dan-feng Zhang, Peng-hui Dou, Dong-xu Zhao, Jing Li, Yu-hong Hu

**Affiliations:** a Department of Gynecology; b Department of Radiotherapy and Chemotherapy; c Department of Physiology, First Affiliated Hospital of Jiamusi University, Jiamusi, China.

**Keywords:** chemotherapy, cisplatin, efficacy, ovarian cancer, randomized controlled trial, safety

## Abstract

**Background:**

Ovarian cancer (OC) is one of the most leading causes of deaths in the Chinese women. The objective of this protocol is to perform a full-scale systematic review on the efficacy of weekly cisplatin (WC) for the treatment of patients with OC.

**Methods:**

Data sources will comprise of PubMed, PsycINFO, Scopus, Opengrey, Cochrane Central Register of Controlled Trials, Embase, Cumulative Index to Nursing and Allied Health Literature, Web of Science, Allied and Complementary Medicine Database, and Chinese Biomedical Literature Database. All relevant randomized controlled trials from searched databases will be identified from their inception to the present. A defined search strategy will be implemented along with eligibility criteria. Relevant data will be extracted according to the predefined data collection form. Methodologic quality will be assessed by using Cochrane risk of bias tool; and data pooled and meta-analysis will be conducted by using fixed-effects, or random-effects model with RevMan 5.3 software.

**Results:**

This proposed systematic review will evaluate the efficacy of WC for patients with OC.

**Conclusion:**

The findings of this study may summarize the latest evidence for the WC on OC.

**Ethics and dissemination:**

Ethical approval is not required for this study, because it will be based on published studies, and existing sources of literature. The results of this study will be disseminated through peer-reviewed journal.

**PROSPERO registration number:**

PROSPERO CRD42018120938.

## 1 Introduction

Ovarian cancer (OC) is one of the most common and lethal gynecologic cancers.^[1–3]^ It has been estimated that about 295,414 new cases and 184,799 deaths occurring in 2018 worldwide.^[4]^ In United States, it accounts for 14,070 deaths and 22,240 new cases in the United States annually.^[5,6]^ In China, it is the 3rd common female cancer.^[7–9]^ It is responsible for 634,000 new cases, 213,000 deaths, and its mortality rate of 3.21 per 100,000.^[10,11]^ This disorder is often diagnosed at an advanced stage because of its asymptomatic characteristics.^[12–14]^ Thus, it still regards as a deadly disease despite significant advances in treatment strategies.^[15]^ Therefore, further study is still required to focus on investigating the pathogenesis and pathophysiology of OC, as well as more effective treatments.^[16]^

Chemotherapy has been widely utilized to treat OC with promising efficacy.^[17]^ It consists of numerous medications, such as cisplatin, carboplatin, fluorouracil, etc.^[18,19]^ Of those, weekly cisplatin (WC) has been used for the treatment of patients with OC effectively and acceptable toxicities.^[20–24]^ However, there is still no systematic review to address its efficacy and safety for patients with OC. Therefore, in this study, we will systematically assess the efficacy and safety for patients with OC.

## 2 Methods and analysis

### 2.1 Eligibility criteria

#### 2.1.1 Types of studies

Only randomized controlled trials (RCTs) comparing the efficacy of WC on OC will be considered. Studies of nonclinical studies, case studies, non-RCTs, and quasi-RCTs will all not be considered.

#### 2.1.2 Types of participants

Participants of all ages with clear clinically diagnosed of OC will be fully considered for inclusion.

#### 2.1.3 Types of interventions

In the experimental group, any types of WC, regardless of dosage, treatment period will all be considered for inclusion. In the control group, any types of therapies can be used, except WC.

#### 2.1.4 Types of outcomes

The primary outcome includes overall survival and overall response rate. The secondary outcomes are the progression-free survival, health-related quality of life, and safety.

### 2.2 Search strategy

A comprehensive search strategy for published studies will be performed using PubMed, PsycINFO, Scopus, Opengrey, Cochrane Central Register of Controlled Trials, EMBASE, Cumulative Index to Nursing and Allied Health Literature, Web of Science, Allied and Complementary Medicine Database, and Chinese Biomedical Literature Database from their inception to the present. A manual search of reference lists provided in relevant studies and reviews will also be conducted to identify any potential studies. The sample search strategy of Cochrane Central Register of Controlled Trials is presented in Table 1. The similar search strategy will also used to other databases.

**Table 1:**
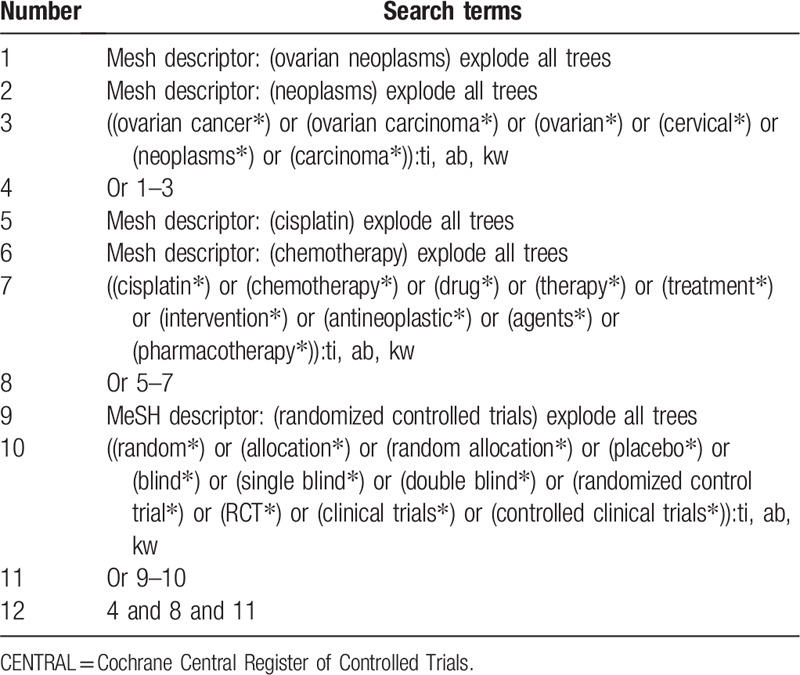
Search strategy applied in CENTRAL database.

### 2.3 Study selection and data extraction

**Figure 1:**
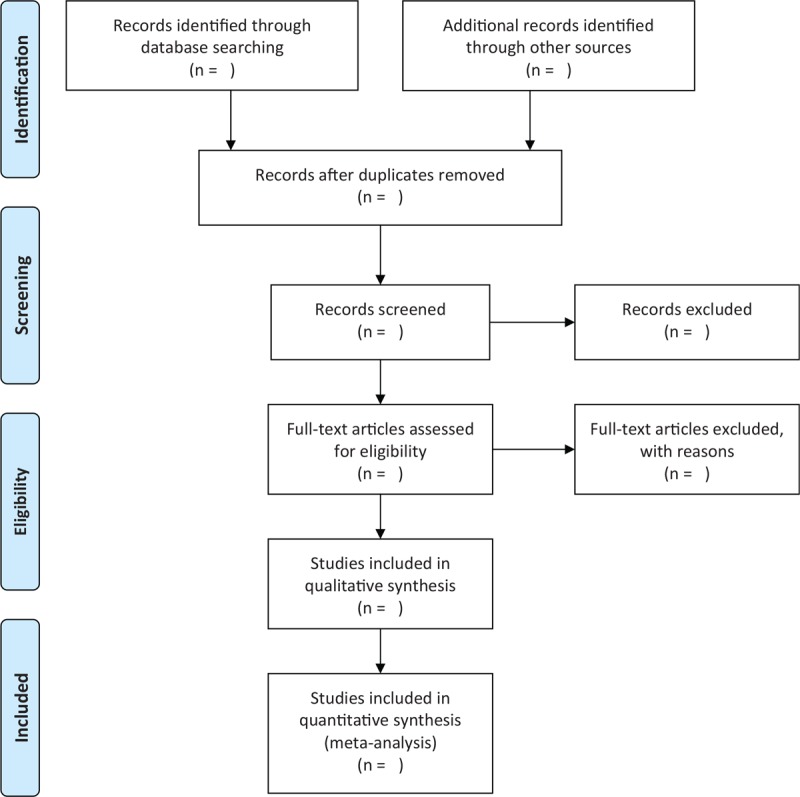
Flowchart of study selection.

Independent screening of the associated titles and abstracts will be conducted with the eligibility criteria. The full-text will also be read if the papers cannot be judged by titles and abstracts alone. All process of study selection follows the Preferred Reporting Items for Systematic Reviews and Meta-Analysis flowchart, and is presented in Figure 1. All study selection will be carried out by 2 authors independently, and a 3rd author will be invited to solve disagreements arise between 2 authors.

### 2.4 Data extraction and management

After study selection, the data will be extracted from each included study by using predefined standard data extracted form. All related data including general information (such as title, 1st author, published year, country, and funding), patient characteristics (such as race, age, gender, number of patients in each group, diagnosed criteria, and inclusion and exclusion criteria), study methods (such as details of randomization, blinding, concealment, and any other potential risk of bias), interventions (such as dosage, frequency, session, and duration), and outcomes (such as primary, secondary outcomes, and safety) will be extracted by 2 independent authors. Any divergences regarding data extraction will be settled down by a 3rd author through discussion.

### 2.5 Missing data dealing with

If insufficient or missing data arise, the primary authors will be contacted by using emails. If we cannot achieve those data, just available data will be pooled and analyzed. In addition, we will also discuss the potential impact of data missing in the discussion section.

### 2.6 Risk of bias assessment

Cochrane risk of bias tool will be utilized to evaluate methodology quality for each included study by 2 independent authors. This tool includes 7 domains and each one is divided as 3 levels of low, unclear, and high risk of bias. Disagreements between 2 authors will be solved by a 3rd author through discussion.

### 2.7 Data synthesis and analysis

RevMan 5.3 software will be applied to pool and to analyze data. Continuous data will be obtained as mean difference and 95% confidence intervals (CIs). Dichotomous data will be obtained as risk ratio and 95% CIs. Heterogeneity will be identified by using *I*^2^ values. The acceptable heterogeneity is considered if *I*^2^ ≤50% and the outcome data will be pooled by using a fixed-effect model. Otherwise, significant heterogeneity is considered, and data will be pooled by using a random-effect model.

### 2.8 Additional analysis

#### 2.8.1 Subgroup analysis

If the heterogeneity is significant, then subgroup analysis will be performed based on the different regions, intervention types, different controls, and outcome tools.

#### 2.8.2 Sensitivity analysis

If it is possible, sensitivity analysis will also be conducted to check the robustness of pooled results data by removing low-quality trials.

#### 2.8.3 Reporting bias

If more than ten eligible studies are included, then funnel plots and Egger regression test will be conducted to detect the possible reporting bias.^[25,26]^

## 3 Discussion

This systematic review is the 1st study to assess the efficacy and safety of WC for patients with OC. We will try our best to search more comprehensive literature sources, including the gray sources to avoid missing any potential studies. All the procedures of study selection, data extraction, and methodologic quality assessment will all be carried out by 2 independent authors. Any disagreements will be settled down with discussion by a 3rd author involved. The results of this study will provide the latest evidence on the efficacy and safety of WC for the treatment of patients with OC. The findings of this study may also provide helpful evidence for clinicians, patients, as well as the future studies.

## Author contributions

**Conceptualization:** Dan-feng Zhang, Peng-hui Dou, Dong-xu Zhao, Jing Li, Yu-hong Hu.

**Data curation:** Dan-feng Zhang, Peng-hui Dou.

**Formal analysis:** Dan-feng Zhang, Jing Li, Yu-hong Hu.

**Funding acquisition:** Dan-feng Zhang.

**Investigation:** Peng-hui Dou.

**Methodology:** Dan-feng Zhang, Dong-xu Zhao, Jing Li, Yu-hong Hu.

**Project administration:** Peng-hui Dou.

**Resources:** Dan-feng Zhang, Dong-xu Zhao, Jing Li, Yu-hong Hu.

**Software:** Dan-feng Zhang, Dong-xu Zhao, Jing Li, Yu-hong Hu.

**Supervision:** Peng-hui Dou, Yu-hong Hu.

**Validation:** Dan-feng Zhang, Peng-hui Dou, Jing Li.

**Visualization:** Peng-hui Dou, Dong-xu Zhao.

**Writing – original draft:** Dan-feng Zhang, Peng-hui Dou, Jing Li, Yu-hong Hu.

**Writing – review & editing:** Dan-feng Zhang, Peng-hui Dou, Dong-xu Zhao, Yu-hong Hu.
